# Particularité de l’anesthésie pour cure chirurgicale du phéochromocytome: à propos d’un cas

**DOI:** 10.11604/pamj.2018.29.31.11156

**Published:** 2018-01-15

**Authors:** Amadou Magagi, Harissou Adamou, Ibrahim Amadou Magagi, Maazou Halidou, Oumarou Habou, Hassane Moussa Diongolé, Maman Sani Rabiou, Mahaman Boukari Baoua

**Affiliations:** 1 Service d’Anesthésie Réanimation de l’Hôpital National de Zinder, Niger; 2 Faculté des Sciences de la Santé de l’Université de Zinder, Niger; 3 Service de Chirurgie Générale de l’Hôpital National de Zinder, Niger; 4 Service d’Urologie de l’Hôpital National de Zinder, Niger; 5 Service de Chirurgie Pédiatrique de l’Hôpital National de Zinder, Niger; 6 Service de Néphrologie de l’Hôpital National de Zinder, Niger; 7 Service de Neurochirurgie de l’Hôpital National de Zinder, Niger; 8 Service d’Anesthésie Réanimation de l’Hôpital National de Zinder, Niger

**Keywords:** anesthésie, chirurgie, phéochromocytome, laparotomie, Niger, Anesthesia, surgery, pheochromocytoma, laparotomy, Niger

## Abstract

Nous rapportons la prise en charge d'une patiente de 37 ans, aux antécédents d'hypertension artérielle (HTA) mal suivie, adressée en consultation chirurgicale avec une symptomatologie faite de lombalgies droites, de vertiges, de céphalées, de sueurs et de palpitations. Durant l'hospitalisation, la tension artérielle (TA) oscillait entre 130/80 mm d'Hg et 190/120 mm d'Hg. Le reste de l'examen clinique ne retrouvait aucune autre anomalie. Le scanner thoraco-abdominal avait montré une masse surrénalienne de 55x45x65 mm comprimant la veine cave inférieure et la veine rénale droite. Le dosage de l'acide vanyl-mandélique urinaire (VMA) donnait un résultat de 11,8mg/24heures. Le dosage des catécholamines sanguin n'a pas été réalisé. Le diagnostic d'un phéochromocytome était retenu et l'indication opératoire était posée. En consultation d'anesthésie l'examen clinique retrouvait un bon état général, une tension artérielle (TA) à 190/120 mmHg, une auscultation cardiopulmonaire sans particularité et un critère prédictif d'intubation non difficile (Mallampati II). La patiente a bénéficiée d'une préparation préopératoire à base d'alpha-bloquants et bêta-bloquants, et d'un inhibiteur calcique. La surrénalectomie a été réalisée par laparotomie médiane. Il n'y avait pas eu d'instabilité hémodynamique lors de la mobilisation et de la résection de la tumeur. Les suites opératoires immédiates ont été simples. Elle a regagné son domicile à J7 postopératoire. Avec un recul de 3 mois, la patiente ne présentait plus de signes cliniques et ses chiffres tensionnels étaient normaux. L'anesthésie pour la chirurgie du phéochromocytome est faisable même en situation de ressources limitées. Une bonne préparation du patient permet d'éviter les complications périopératoires.

## Abstract (en)

This study decribes the treatment of a 37-year old female patient, with a history of poorly treated arterial hypertension (AH), referred to surgical consultation with right lower back pain, vertigo, headache, sweating and palpitations. During hospitalization, blood pressure (BP) ranged between 130/80 mm Hg and 190/120 mm Hg. The remainder of the clinical examination was normal. Thoracoabdominal scanner showed adrenal mass measuring 55x45x65 mm compressing the inferior vena cava and the right renal vein. The assay of urinary vanilmandelic acid (VMA) was 11.8mg/24hours. The assay of catecholamines in the blood was not performed. The diagnosis of pheochromocytoma was retained and surgical indication was determined. During pre-anaesthesia consultation, clinical examination showed good general condition, blood pressure (BP) to190/120 mmHg, normal cardiopulmonary auscultation and prediction criterion of easy intubation (Mallampati II). The patient underwent preoperative treatment with alpha-blockers and beta-blockers, and calcium channel blocker. Adrenalectomy was performed using midline laparotomy. The patient didn?t have hemodynamic instability during mobilization and tumor resection. The postoperative course was uneventful. She was discharged home on postoperative day 7. At 3 months? follow-up, the patient had no clinical signs and blood pressure was normal. Anesthesia for the surgical treatment of pheochromocytoma is feasible even in resource-limited settings. An adequate preoperative preparation of the patient helps prevent complications.

## Introduction

Le phéochromocytome (PCC) est une tumeur neuroendocrine rare développée aux dépens des cellules chromaffines des glandes surrénales sécrétant des catécholamines [1-5]. Elle est à l'origine de 0,05 à 0,6% des hypertensions artérielles (HTA) permanentes [1,2,5,6]. La découverte du phéochromocytome peut être fortuite dans 15 à 20% de cas [2,7]. L'HTA due à cette affection est curable chirurgicalement plus souvent par la laparoscopie que par la laparotomie [2,3,5,7-9]. Le PCC représente un défi important pour l'anesthésiste-réanimateur. En effet, un risque d'instabilité hémodynamique demeure même après une anesthésie adaptée du fait de la libération des catécholamines [1,5]. Nous rapportons l'observation d'une patiente opérée sous anesthésie générale pour phéochromocytome et discutons avec les données de la littérature.

## Patient et observation

Une patiente paucigeste, âgée de 37 ans, pesant 70 kg et mesurant 170 cm, aux antécédents d'hypertension artérielle (HTA) mal suivie était adressée en consultation chirurgicale avec une symptomatologie faite de lombalgies droites, de vertiges, de céphalées de sueurs et de palpitations, évoluant depuis plusieurs mois. Durant l'hospitalisation, la tension artérielle (TA) oscillait entre 130/80 mm de Hg et 1^9^0/120 mm de Hg. Le reste de l'examen ne retrouvait aucune autre anomalie. Le scanner thoraco-abdominal montrait une masse surrénalienne de 55x45x50 mm comprimant la veine cave inférieure et la veine rénale droite. Le dosage de l´acide vanyl-mandélique urinaire (VMA) objectivait 11,8mg/24heures. Le dosage des catécholamines n´a pas été réalisé. Le diagnostic d'un phéochromocytome était retenu et l'indication opératoire était posée. En consultation d'anesthésie, l'examen clinique retrouvait un bon état général, une tension artérielle (TA) à 190/120 mmHg, une auscultation cardiopulmonaire sans particularité et un critère prédictif d'intubation non difficile (Mallampati II). La patiente était classée ASA III. Devant les chiffres élevés de la TA la patiente fut mise sous Nicardipine 20 mg à raison de 1cp x 3/ j, hydroxyzine 25 mg à raison de 1cp le soir au couché, et propranolol 10mg x 2/j. Une préparation colique associant régime sans résidus et laxatifs était faite à la patiente.

En salle opératoire, la Nicardipine 1mg /ml en seringue électrique était utilisée. Le furosémide 40 mg, l'Esmolol 250mg/ml dilué à 12,5mg/ml et la dopamine en seringue électrique étaient préparé à raison de 10 mg/ml/h. L'induction anesthésique était faite avec de l'etomidate à raison de 20mg, du vécuronium 4mg, et du fentanyl 50 µg. L'intubation a été facile avec une sonde 7,0 CH avec ballonnet. L'entretien anesthésique associait le fentanyl, l'isoflurane, le midazolam et le vécuronium. La gestion de l'hémodynamique était faite avec du Nicardipine 1mg/ml en seringue électrique, du furosémide 40mg et de la dopamine 10mg /ml/h en seringue électrique. Durant l'intervention les chiffres tensionnels oscillaient entre 119 / 110 et 130 / 80 mm de Hg. Le monitorage non invasif de la pression artérielle (PNI), de la saturation en oxygène (SPO2), de la capnométrie (ETCO2), de l'électrocardiogramme et de la diurèse était fait. La surrénalectomie était réalisée par laparotomie médiane par incision sus et sous ombilicale avec résection de l'appendice xiphoïde. L'ouverture du péritoine pariétal postérieur et la libération de l'angle colique droit avaient permis de récliner les veines cave inférieure et rénale droite, et d'aborder la surrénale droite. Le contrôle vasculaire et la surrénalectomie droite étaient effectués. Il n'y avait pas eu d'instabilité hémodynamique lors de la mobilisation et de la résection de la tumeur. Les suites opératoires immédiates ont été simples avec une stabilité hémodynamique et ventilatoire. A la quarante huitième (48) heures postopératoire, la patiente a été transférée au service de chirurgie pour la suite du traitement. Elle a regagné son domicile à J7 postopératoire. Avec un recul de 3 mois, la patiente ne présentait plus de signes cliniques et ses chiffres tensionnels étaient normaux.

## Discussion

Les phéochromocytomes sont des tumeurs neuroendocrines rares développées aux dépens des cellules chromatines du système nerveux parasympathique et sympathique [1-4,8,10-12]. Ces cellules produisent de l'épinéphrine, de la norépinéphrine et de la dopamine et les symptômes du phéochromocytome (poussées hypertensives, céphalées, sueurs, dysrythmies, accidents vasculaires cérébraux, ischémies myocardiques) résultent de la libération incontrôlée de ces substances [2,7,8,13]. Les paragangliomes sont dérivés embryologiquement de la crête neurale [10,11]. Ce sont des tumeurs bénignes dans 90% de cas, mais très bien vascularisées [11,12]. Ils peuvent être fonctionnels et sécréter des catécholamines; on parle dans ce cas, de phéochromocytome lorsque la tumeur est développée aux dépens de la médullosurrénale [4,5,7,8,12]. Les paragangliomes fonctionnels peuvent se situer au niveau thoracoabdominopelvien, dans le médiastin et/ou le rétropéritoine (ou phéochromocytomes ectopiques) [1,8,11,12]. Les paragangliomes non sécrétants, sont moins symptomatiques et se localisent le plus souvent au niveau de la tête et du cou (glomus carotidien, glomus tympanique, glomus jugulaire, glomus vagal, etc.) [8,11,12]. Du fait d'un nombre important de cas méconnus, asymptomatiques ou de présentations cliniques très variables, l'épidémiologie des phéochromocytomes reste difficile à établir [12-13]. Il s'agit d'affection rare dont la prévalence est probablement sous-estimée [12]. La prévalence des phéochromocytomes est de 0,05 à 0,6% parmi les patients hypertendus [1,3,7,12]. L'incidence à l'échelle de la population générale est estimée à 1 nouveau cas pour 100.000 personnes par année [1-3,12]. Ces données sont sous-estimées si l'on tient compte des résultats de séries autopsiques, où la prévalence des PCC atteint 1 pour 2000 [6]. Chez l'adulte, 90% des tumeurs sont de localisation médullo-surrénalienne alors qu'un tiers chez l'enfant est de localisationextra-surrénalienne. On les trouve alors au niveau des reliquats embryologiques de la crête neurale [1,5-7,12].

Le diagnostic du PCC n'est pas toujours facile. Dans la majorité des cas, le PCC est suspectée devant une symptomatologie peu spécifique où domine l'hypertension artérielle, la sueur, les palpitations, les céphalées [12-14]. L'HTA du PCC est caractérisée par une instabilité importante. Elle est paroxystique uniquement dans 50% des cas, permanente dans 29 % et inexistante dans 13% des cas [1,5,12]. Le diagnostic peut également être évoqué devant un syndrome de masse entraînant des douleurs abdominales, ou à la suite de la découverte de métastases [12]. Le diagnostic biologique repose sur les dosages plasmatiques et urinaires des dérivés méthoxylés (métanéphrine, normétanéphrine) qui peuvent être élevés [1,5,10,12,13]. Les examens de localisations de 1^ère^ intention sont la tomodensitométrie ou l'imagerie par résonnance magnétique. C'est une tumeur arrondie, située en position surrénalienne au niveau du pole supérieur du rein, séparée de celui-ci par un plan de clivage net et développée aux dépens de la médullo- surrénale. Sa taille est variable d'une petite prune à un pamplemousse pouvant atteindre 1,5 kg. Cette tumeur est richement vascularisée par des pédicules artériels issus des artères rénales ou de l'aorte. L'un des temps opératoire à risque sera la dissection de la tumeur et la ligature successive des différents pédicules veineux et artériels [3,12]. Une fois le diagnostic de PCC posée, l'interrogatoire doit rechercher des arguments en faveur d'une maladie génétique par la réalisation d'un arbre génétique [11-13]. La recherche d'anomalies thyroïdiennes, parathyroïdiennes, dermatologiques, oculaires, pancréatiques et/ou rénales doit être systématique [11-13]. En plus, la recherche de mutations germinales doit être systématiquement proposée [11,12]. La forme familiale est retrouvée dans environ 10-30% des cas, on parle alors de paragangliome héréditaire. Elle peut être isolée ou s'intégrer dans des syndromes génétiques composites : les néoplasies endocriniennes multiples (NEM de type IIa et IIb) [11,12].

Le traitement standard du phéochromocytome est la résection chirurgicale [3-5,12]. Il est important de ne pas méconnaître cette pathologie, car elle fait partie des rares causes curables d'hypertension artérielle [1-5,8]. La surrénalectomie par voie laparoscopique rétropéritonéale ou transpéritonéale reste le « gold standard » pour la résection des PCC de moins 6 cm de diamètre et pesant moins de 100g [1-3,5,8]. Les résultats en terme de temps opératoires, de complications per- ou postopératoires pour ces abords sont similaires [1-3,5,8]. Le traitement chirurgical du phéochromocytome consistait encore il y a une vingtaine d'années en une large laparotomie [3,5]. Aujourd'hui, la laparoscopie est devenue la voie d'abord de référence pour la chirurgie du PCC, et la laparotomie est réservée aux tumeurs supérieur à 8 cm, à l'existence d'une fibrose périsurrénalienne, de lésions récidivantes ou de difficultés d'hémostase [1,2,5,15]. D'autres facteurs comme l'expérience du chirurgien, la convenance et la préférence sont des éléments à prendre en compte dans l'indication de la laparoscopie. Notre patiente était opérée par laparotomie pour des raisons techniques. En effet, les chirurgiens n'avait pas l'expérience de cette chirurgie par voie coelioscopique d'une part et il n'existait pas de colonne de cœlioscopie disponible d'autres part. La prise en charge du PCC constitue un défi anesthésiologique en raison des caractéristiques cliniques de cette affection et leurs implications [1,5,7,10,13,14]. D'un point de vue hémodynamique, la situation est complexe, en particulier lorsque le PCC est méconnue et est découvert fortuitement en peropératoire où il expose des situations potentiellement mortelles [2,3,9,10,13-15]. Rousson et al [14] la considère comme le ‘cauchemar’ de l'anesthésiste lorsque la découverte du PCC est fortuite en peropératoire. Le diagnostic, l´évaluation préopératoire de ces patients est la clé du succès de la prise en charge périopératoire [1-3,7,9,15].

Le but de la préparation préopératoire adéquate est de prévenir une poussée hypertensive aiguë en salle opératoire, mais aussi de minimiser les instabilités hémodynamiques induits par la libération des catécholamines pendant l'anesthésie et la chirurgie [1,5,9,10,13]. La manipulation du PCC lors de sa dissection et son ablation chirurgicale peut s'accompagner d'importantes variations hémodynamiques (HTA et des arythmies sévères). En effet pendant la dissection d'énorme quantité de catécholamines sont déversées dans la circulation. Le taux plasmatique peut être multiplié par 200 nécessitant parfois l'interruption momentanée de l'acte chirurgical. Les praticiens prenant en charge ces patients doivent s'attendre à gérer l'instabilité hémodynamique pouvant survenir à tout moment [1,2,5,7-9]. Ces perturbations qui peuvent se produire lors des manipulations chirurgicales peuvent être contrôlées par l'utilisation pré et per opératoire des drogues tels que les alpha-bloquants, les bêta-bloquants, les inhibiteurs calciques notamment la nicardipine, le nitropruside, la nitroglycérine, le magnésium et/ou par l´approfondissement de l´anesthésie [1,3,7,9,10,13]. Le sulfate de magnésium est facilement disponible, bon marché, sûr et efficace pour le contrôle hémodynamique avant la résection tumorale. Il a démontré son´efficacité chez les adultes, les enfants et dans des scénarios plus rares, tels que la résection du phéochromocytome pendant la grossesse [10,13]. Une bonne collaboration entre les chirurgiens et les anesthésiologistes et les soins postopératoires diminuent le taux de complications et améliorent le résultat [1,8,9]. La prise en charge du PCC est multidisciplinaire où le chirurgien procède à l'ablation de la tumeur, l'anesthésiste assure la stabilité hémodynamique périopératoire et le cardiologue et endocrinologue gèrent l'aspect cardiométabolique [1,8,9]. Pour Billard et al [9], le contrôle de la TA préopératoire par alpha-bloquant ou inhibiteur calcique est fondamental avant le geste chirurgical. Il doit être associé à un remplissage massif, une utilisation de drogues anesthésiques réversibles, de traitements symptomatiques (par exemple : rémifentanil, sévoflurane, inhibiteur calcique et esmolol) et d'une surveillance postopératoire étroite et prolongée [3,8-10,13]. Selon les critères de Roizen (1982) rapportés par Ramakrishna et al [1], pour évaluer l'efficacité du traitement par alpha-bloquants, différents critères doivent être réunis : pas de TA > 160/90mm de Hg pendant 24 heures avant la chirurgie ; pas d'hypotension orthostatique avec des chiffres de la TA < 80/45 mm de Hg ; aucune modification de l'onde ST ou T une semaine avant la chirurgie et pas plus de 5 contractions ventriculaires prématurées par minute [1].

Sur le plan physiopathologique, le contrôle de l'HTA avant la chirurgie a pour objectif de remédier aux symptômes inconfortables (céphalées, palpitations), de diminuer les complications hémorragiques ou cardiovasculaires et de restaurer une volémie efficace réduite par la vasoconstriction chronique due aux catécholamines [1,5,7]. Sur le plan pronostic, une HTA en préopératoire est un facteur de complications et de mortalité [1,9,13]. Il y a environ 50-70 ans, la mortalité périopératoire associée aux premières surrénalectomies pour phéochromocytome était de 25 à 40 % [2,5,8]. Aujourd'hui, avec l'amélioration des techniques anesthésiques, la chirurgie du phéochromocytome est associée à une morbidité post-opératoire faible [1,3,5,9,15]. La morbidité chez les patients souffrant de phéochromocytome comprend les complications chirurgicales, l´hypertension sévère intra-opératoire et l´effondrement hémodynamique nécessitant un soutien vasopressif et une ventilation mécanique après l´élimination de la tumeur [1,3,8,9]. Les grandes tumeurs sont associées à des fluctuations hémodynamiques plus importantes au cours de la chirurgie pour le phéochromocytome [5,15]. Cependant, cela n´est pas associé à des résultats globaux défavorables [15]. Le risque de récurrence de cette tumeur n'est pas exclu même après une résection complète [3,9,12].

## Conclusion

L'anesthésie pour phéochromocytome constitue encore un challenge à cause de la sécrétion incontrôlée de catécholamines. La prise en charge chirurgicale nécessite une approche pluridisciplinaire pour lutter contre la morbi-mortalité périopératoire. L'anesthésie pour la chirurgie du phéochromocytome est faisable même en situation de ressources limitées. Une meilleure connaissance de la physiopathologie et des effets pharmacologiques des médicaments, une préparation préopératoire optimale et méthodique, permettent à l'anesthésiste et aux autres praticiens de répondre aux défis de la prise en charge du phéochromocytome.

## Conflits d’intérêts

Les auteurs ne déclarent aucun conflit d'intérêts.
